# Providing care to people on social assistance: how dentists in Montreal, Canada, respond to organisational, biomedical, and financial challenges

**DOI:** 10.1186/1472-6963-14-472

**Published:** 2014-10-10

**Authors:** Christophe Bedos, Christine Loignon, Anne Landry, Lucie Richard, Paul J Allison

**Affiliations:** Division of Oral Health and Society, Faculty of Dentistry, McGill University, 2001 McGill college avenue, Montreal, Quebec H3A1G1 Canada; School of Public Health, Université de Montréal, Montréal, Quebec Canada; Institut de Recherche en Santé Publique de l’Université de Montréal (IRSPUM), Montréal, Quebec Canada; Faculty of Medicine, University of Sherbrooke, 150 Place Charles Lemoyne Bureau 200, Longueuil, Quebec J4K 0A8 Canada; Direction de la Santé Publique, Agence de la Santé et des Services Sociaux de Montréal, 1301, rue Sherbrooke Est, Montréal, Québec H2L 1M3 Canada; Institut de Recherche en Santé Publique de l’Université de Montréal (IRSPUM), Faculté des sciences infirmières, Université de Montréal, C.P. 6128 Succursale Centre-ville, Montréal, Québec H3C 3J7 Canada

**Keywords:** Poverty, People on social assistance, Qualitative research, Access to dental services, Dentist-patient relationship, Discrimination

## Abstract

**Background:**

Dentists report facing difficulties and experiencing frustrations with people on social assistance, one of the social groups with the most dental needs. Scientists ignore how they deal with these difficulties and whether they are able to overcome them. Our objective was to understand how dentists deal with critical issues encountered with people on social assistance.

**Methods:**

We conducted in-depth, semi-structured interviews with 33 dentists practicing in Montreal, Canada. The interview guides included questions on dentists’ experiences with people on social assistance and potential strategies developed for this group of people. Analyses consisted of interview debriefing, transcript coding, and data interpretation.

**Results:**

Dentists described strategies to resolve three critical issues: missed appointments (organisational issue); difficulty in performing non-covered treatments (biomedical issue); and low government fees (financial issue). With respect to missed appointments, dentists developed strategies to maximise attendance, such as motivating their patients, and to minimise the impact of non-attendance, like booking two people at the same time. With respect to biomedical and financial issues, dentists did not find any satisfactory solutions and considered that it was the government’s duty to resolve them. Overall, dentists seem reluctant to exclude people on social assistance but develop solutions that may discriminate against them.

**Conclusions:**

The efforts and failures experienced by dentists with people on social assistance should encourage us to rethink how dental services are provided and financed.

## Background

People on social assistance are among the poorest members of North American societies. Unfortunately, they experience high levels of oral illness and, despite the benefit of public dental coverage, rarely consult dentists [1–5]. People on social assistance have reported various difficulties in accessing dental clinics that could explain this situation. In particular, some have expressed experiencing non-welcoming attitudes and even discriminatory practices from dental professionals [6–8].

In parallel, dentists have reported high levels of frustration with people on social assistance [9–11]. In a recent article, we showed that frustrations and challenges experienced by Canadian dentists fall into the following 3 categories [12]: 1) organizational issues, that dentists related to frequent “missed appointments” that upset their schedule; 2) biomedical issues, referring to dentists’ difficulties in performing treatments not covered by public dental insurance; and 3) financial issues, as people on social assistance would not generate much income, mainly due to low fees.

This being said, we still ignore how dentists adapt to these three kinds of difficulties. We know that some dental professionals systematically exclude people on social assistance, but we ignore if others are able to develop solutions to overcome those difficulties. Consequently, the objective of this article, which is part of the study mentioned before [12], was to understand how dentists deal with problems encountered with people on social assistance. More specifically, we wanted to know the kinds of strategies that dentists developed with respect to organisational, biomedical, and financial issues associated with people on social assistance.

## Methods

### Research design

In order to obtain an in-depth understanding of dentists’ perspectives and experiences with people on social assistance, we used a descriptive qualitative research design. It was based on open-ended, semi-structured interviews with dentists that were conducted simultaneously with data analysis. It is important to mention that we already described our methodological approach in a recent article, and we invite the readers to refer to the latter.

### Sampling strategy

This study was conducted in Montreal, a Canadian city with around 170,000 adults on social assistance and their dependents [13]. It also counts almost 1400 general dental practitioners who, for the great majority, work in fee-for-service private clinics [14]. It is important to note that Canada has a publicly funded health care system that does not cover curative dental services. Most Canadian provinces have developed dental care insurance programs though, but these programs remain limited in scope. In the province of Quebec, for instance, only children less than 10 years of age and people on social assistance (and their dependants) benefit from such a dental insurance. It covers most basic dental care, such as routine visits, radiographs, amalgam restorations, dental extractions and even dentures, but excludes more expensive treatments such as endodontics (root canal therapy) and prosthetic crowns [15].

We used a maximum variation sampling strategy [16] to recruit general dentists with various levels of professional exposure to poverty. We thus selected professionals practicing in different types of neighbourhoods, sending them a written invitation, then telephoning them to plan an interview. We stopped recruiting when we obtained data saturation, “the point at which additional data does not improve understanding of the phenomenon under study” [17].

### Data collection

Three experienced interviewers with different backgrounds (sociology, anthropology, and public health) collected data between 2004 and 2007. They conducted in French language in-depth, semi-structured interviews. Most were organised in dentists’ offices and lasted between 60 and 120 minutes; they were audio-recorded and then transcribed verbatim. Before the interview, dentists signed a consent form that was approved by the academic ethics committee of McGill University’s Faculty of Medicine. Using an interview guide, researchers focused on dentists’ experiences with people on social assistance. In particular, they tried to identify the difficulties faced by dentists, and to better understand how they responded to these issues. The interviewers thus invited dentists to freely describe the strategies and solutions they used to resolve problems associated with people on social assistance.

### Data analysis

We conducted a thematic analysis that Braun and Clarke describes as a “method for identifying, analysing and reporting patterns (themes) within data” [18]. After each interview, the interviewer and the main researcher conducted debriefings, based on notes written by the interviewer during and after each interview; these debriefings served to evaluate the data collection, summarise major findings, present emerging hypotheses, and prepare the following interviews. We then coded the interview transcripts with NVivo software: starting with an initial list of codes inspired by the research questions, we refined this list throughout the coding; this process involved cutting the transcripts into meaningful segments and assigning codes to the segments. We then regrouped the codes into wide themes and displayed them in analytic matrices [19]. We finally wrote texts that described the themes and illustrated them with data extracts. To improve the rigor and credibility of our findings, three members of the research team conducted this process, checking and validating their interpretations.

### Description of the sample

The sample is composed of 33 dentists, including 21 men and 12 women (Table 1). It is diverse in terms of dentists’ age, which ranged from 26 to 70 years, cultural background, type of clinical practice, and professional status.

**Table 1:** **Description of the sample (N = 33 dentists)**

Categories	N
Age	
21-30	6
31-40	8
41-50	9
51-60	5
61+	5
Gender	
Female	12
Male	21
Cultural background	
Western background (Canadian)	18
Non-western background (Non-Canadian)	15
Years of experience as a dentist	
0-5	2
6-15	14
16-30	9
31+	8
Type of clinical setting	
Multi practice	21
Solo practice	12
Professional status	
Owner (or co-owner)	25
Employed (paid by percentage)	8

## Results

In the following paragraphs, we will describe the solutions and strategies developed by dentists with respect to each of the 3 categories: organisational, biomedical, and financial. We will then describe the situations that influence dentists’ decision to exclude or not a person on social assistance.

### Organisational issues: how dentists deal with missed appointments

With respect to missed appointments, dentists adopted strategies that we classified under two main categories: 1) Maximising attendance; and 2) Minimising the impact of non-attendance (Table 2).

**Table 2:** **Strategies developed by dentists with respect to organisational, biomedical, and financial issues associated with people on social assistance**

Types of issue	Solutions developed by dentists
Organisational: how dentists deal with missed appointments	1) Maximising attendance of people on social assistance:
	a) Motivating and/or threatening people
	b) Finding time periods when people are available
	c) Using “reminder” strategies
	d) Avoiding planning sessions in advance and using people as “fillers”
	2) Minimising the impact of non-attendance:
	a) Booking appointments at times of the day/week that reduce disruption to a minimum
	b) Shortening clinical sessions and/or double-booking
Biomedical: how dentists deal with treatments not covered by public dental insurance	1) Limiting the therapeutic options to covered services
	2) Encouraging people on social assistance to accept non-covered treatment (at their own cost)
	3) Performing non-covered treatment at reduced cost or for free
Financial: how dentists deal with the low fees of public dental insurance	1) Avoiding performing covered treatments that offer only a low profit margin
	2) Reducing expenses of the dental clinic

As mentioned in Table 2, dentists tried to maximize attendance in 4 different ways: a) Motivating and sometimes threatening people; b) Finding appropriate time period for the appointments; c) Using reminder strategies; d) Avoiding planning sessions in advance and using people on social assistance as “fillers”.

The first strategy for maximising attendance consisted of “motivating” people and emphasizing the importance of respecting the schedule. Dentists sometimes reinforced their message with two threats: the first was to ask people to pay a fee each time they missed an appointment; the other, more radical, was to “close their file” and invite them to look for another dentist.

*“Listen, you have to sign a contract. If you miss an appointment, it’s $35.” There are people on social assistance who refuse to pay that.* [Translation] [CL17]

*We’ve already threatened people that we’ll close their file, that they’ll have to find another dentist. This actually does the trick.* [Translation] [CB1]

Another strategy for maximising attendance was to find times that would suit people on social assistance best. For instance, dentists assumed that the former tended to wake up late because of not having a steady job; consequently, they avoided booking them early mornings and rather favoured the middle of the day.

Participant*: There are those who we know don’t get up until 11 in the morning, for example, so we don’t give them an 8 am appointment.*

Interviewer*: At 9 am?*

Participant*: Forget that. In fact we fit them in at 1 in the afternoon.* [Translation] [CL8]

*So for the appointments, I find it works much better at the end of the morning. No early morning appointments. Or the beginning of the afternoon.* [Translation] [CL15]

Dentists described a third strategy that consisted of reminding people to attend, generally through a phone call the day before. One dentist mentioned that her secretary would even call people several times, including on the day of the appointment, but deplored that this was not always successful.

*As for the others, we also call them on the same day and for them, I also know that the receptionists in* [a second office]*, they also put pressure on them. They say, “Here, listen, you’ve missed several appointments already. You really need to come in.” But even then, it doesn’t work for everybody.* [Translation] [CL11]

When the three previous strategies failed, dentists became reluctant to give appointments in advance. Some consequently explained that they had drawn up a list of people “on call”: when a time slot became free on their agenda, they contacted people on this list and offered them an appointment the same day. Participants also invited these patients to call them when they were ready to come, and see if they were able to book them in at short notice. In this way, people on social assistance became “fillers” in dentists’ agendas.

*So, we set six-monthly appointments. For someone on social assistance, we decided we wouldn’t do that. That will cost us a stamp, that will cost us a time slot, that will cost us a missed appointment, a phone call. No, no. We stopped that.* [Translation] [CL7]

*That’s twice he’s missed his appointment. It’s the receptionist who says, “I’m starting to get fed up with being jerked around”.* […] *So we don’t call them anymore or we see them in emergency. [The receptionist] says, “You’ll come when you’ve got a broken tooth”.* [Translation] [CL18]

*It’s happened that I’ve closed files, or a particular patient keeps missing appointments but he absolutely wants to come back and see you, and everything. […] So we’ll say, “Look, we’re not going to give you any more appointments in advance. You have to call us the same morning.”* [Translation] [CL6]

In addition to describing ways to maximise attendance, participants presented two strategies to minimise the impact of missed appointments on their schedule: a) Booking appointments at times that would reduce potential disruption; and b) Shortening clinical sessions and/or “double-booking”.

The first strategy, as a participant explained, was to book people on social assistance at the end of the working day, so that “*if they do not come,* [she] *can leave.*” [CB1] Another dentists further clarified: “*We don’t offer them the best spots – the evening appointments, Saturdays, when we work Saturdays. We try to reserve those times for people who pay for themselves.”* [Translation] [CL7]

The second strategy was to plan shorter appointments in order to reduce the time lost if the person did not show up. A version of this strategy was what participants named “double-booking”: it consisted of booking two people at the same time and so increasing the chances of having at least one present. This being said, dentists remained reluctant to do so, due to the stress that would occur if two patients showed up at the same time.

Interviewer: *Do patients on social assistance usually have long appointments?*

Participant*: Usually we give fewer [long appointments]. No. We try to schedule several appointments.*

Interviewer*: Shorter ones?*

Participant*: Yes*

Interviewer*: Why is that?*

Participant*: Because we think they’ll come less to appointments.* [Translation] [CB1]

*Sometimes we might even double-book them, you know, with another patient. If the other patient is also a risk, we book both together.* […] *If both of them show up, then it’s us who’ll be a bit behind but the chances are that only one of them will show up, so it’ll be OK.”* [Translation] [CL2]

It is important to note that dentists could also apply these strategies to people not on social assistance. However, they made a distinction between people on social assistance and “regular people” who consulted for the first time: whereas they would give the benefit of the doubt to “regular people”, they would often suspect people on social assistance of being lazy, and therefore adopt one or several of these strategies. For instance, a dentist explained that, *“We don’t give them a very long appointment. Especially when we don’t know them.”* [CL7] However, once the dentists better knew the attitudes and practices of a person on social assistance, they could reconsider the pertinence of using these strategies.

### Biomedical issues: how dentists deal with treatments not covered by public dental insurance

Dentists dealt with these difficulties in three main ways that we will describe in the following paragraphs: 1) Limiting the therapeutic options to services that are covered; 2) Encouraging people on social assistance to pay for non-covered treatments; and 3) Performing non-covered treatments for free or at a reduced cost (Table 2).

With the first strategy, dentists considered that discussing non-covered therapeutic options with people on social assistance was a waste of time. Consequently, they would suggest treatment plans that expunged non-covered services, and by doing so adopting a practice that they designed as basic dentistry, and sometimes “dentistry for the poor”.

*So if you’re going, say, to see a doctor because you’re suffering with a pain in your heart, he’s going to do some tests, he’s going to take a look. He’s not going to say, “She needs a pacemaker but she can’t afford it. I have nothing more to say to her”. Whereas the dentist will do that. If the patient comes in and he knows she’s on social assistance, he won’t spend half an hour telling her the tooth needs a crown. That’s just a waste of time.* [Translation] [CL2]

Facing ethical dilemmas, especially when they considered that a tooth should be restored instead of extracted, dentists reported offering wider therapeutic options to some people on social assistance. They adopted this strategy when they knew the patients well or considered that, because of their young age or high level of motivation, they deserved better quality care. In such cases, dentists would encourage people to undergo non-covered treatments, and sometimes provide incentives, such as offering them to pay by instalments.

*We said, “Look, you’ll need to make three payments for your root canal work, I’m able to do my part. I know that* […] *we can tell that you may not have the money to pay for it all at once.” We said, “Look, you can pay it in three instalments.”* [Translation] [CL6]

This said, the ability of people on social assistance to pay was so low that some dentists would sometimes adopt a last resort strategy: treat them for a reduced fee or even for free. Dentists acknowledged, however, that this constituted an occasional practice that could not be generalised because of its financial implications.

*I told him that it’s not something that’s covered, but I was willing to do it just to give him a helping hand. If all my patients were like that, I couldn’t do it, but if I get one from time to time, I can do it, yes.* [Translation] [CL1]

*It happened with some patients of mine, I had two patients who were on social assistance, who had been coming to me for a long time, and for whom I did root canal work free of charge, because I didn’t want to extract the tooth - it would have really upset me.* [Translation] [CL5]

### Financial issues: how dentists deal with low fees

Dentists reported two strategies to deal with the low governmental fees for people on social assistance: 1) Avoiding performing covered treatments that provided a particularly little profit margin; 2) Reducing their overall expenses (Table 2).

With respect to the first strategy, several dentists expressed their reluctance to make prostheses, which would not be profitable enough. They did not necessarily refuse to undertake them, but acknowledged that they did not encourage people on social assistance to choose them, unless they were “good patients”.

*There’s no longer much profit in it. So, with regard to prosthetics, let’s say we don’t chase people. If I have good patients who want a prosthetic device, I do it. But I don’t run after patients to offer them prosthetics if they’re on social assistance* [Translation] [CL8]

*The rates that are currently paid don’t allow us to make partials for those on social assistance. I can’t do it. My lab charges me more than the [government] gives me for making a partial.* [Translation] [CL1]

The other strategy, which was described by participants delivering care in underprivileged neighbourhoods, consisted of reducing the overall expenditures of their dental office. This would involve limiting the number of employees, restricting the costs for equipment and materials, and even reducing the number of visits necessary to perform a treatment.

*We adjust to it. As I said, I have less staff, I cut the staff, and that’s pretty much it.* […] *I don’t cut back on the quality of materials. But I negotiate with those who sell the materials to give me the best price possible. […] In the end, all spending is justified. We don’t spend on luxuries. No, there are no luxury items. All spending is carefully monitored.* [Translation] [AL4]:

### When the strategies fail: toward the exclusion of people on social assistance

Even though all dentists reported encountering difficulties with people on social assistance, none mentioned having adopted a policy that *purposefully* and *systematically* excluded them; they acknowledged, though, that this approach existed in the dental community. Their reasons for accepting treating people on social assistance were varied: many explained that it was their professional duty to treat everyone in the society, some even expressing compassionate thoughts about people living in poverty. Others reported more pragmatic reasons, explaining that young dentists needed people on social assistance to gain clinical experience and develop their dental practice.

Dentists thus decided to exclude people on social assistance on an individual basis only, the main reason being missed appointments. Their decision, however, was modulated by the way they perceived poverty. Dentists working in underprivileged areas tended to express indulgence and empathy for people who faced hard and challenging lives. Others, on the contrary, were inclined to blame patients on social assistance for their way of life and attributed non-attendance to laziness and neglect. In such cases, dentists considered excluding a patient as a legitimate option and would do so very clearly:

*Yes, patients like that, I’m happy to be rid of them, I make no effort to keep them. None. It’s their choice. If you don’t respect me, go elsewhere. It’s as simple as that.* [Translation] [AL5]

Others dentists preferred a more subtle method to exclude people, for instance by stopping phone call reminders for routine visits; for more urgent care, they would offer appointments only in the long term, thus expecting the person to consult another dental clinic.

*Once they’ve missed two, three appointments, you start to be more tactical and say, “We don’t have any free spots for three months.” No, but it’s true.* [Translation] [CL6]

Biomedical issues could also trigger the exclusion of people on social assistance. Confronted with the decision to extract a tooth that, according to them, could be restored, some dentists preferred referring a person to another dentist rather than performing a treatment that would contradict their ethical values.

*I let him leave, and I referred him to another dentist in the clinic who would likely have no problem in removing it.* [Translation] [CL11]

In the same perspective, several dentists carried out a form of clinical dentistry that *de facto* excluded people on social assistance. This type of dentistry aimed at providing “high quality” services and was based on “high tech” approaches. It included cosmetic treatments and use of implants, for instance, which were not covered by public dental insurance. As an example, some dentists explained that they no longer used amalgam, preferring composites, materials that are not covered for posterior teeth by the public dental insurance.

It is interesting to note that, conscious of this *de facto* exclusion, some dentists developed alternate solutions. For instance, one explained that he made an arrangement with his associate in order to welcome publicly covered people and thus respond to the needs of the community. Whereas one dentist would provide “sophisticated” treatments to a particular clientele, excluding children and people on social assistance, his partner, located in the same office, would welcome the latter.

*In other words, I abandoned public dental insurance patients completely. I didn’t withdraw from public dental insurance, but I made a choice, a preference, that my partner would see this clientele. This would give me more time to see my clientele. My clientele, in every case, the clientele that I had identified as a clientele needing treatments that I had, I had learned that was a little more sophisticated. Do you see? This is not only about implant dentistry, these are more advanced prosthetic dentistry cases.* [Translation] [CL9]

Finally, financial issues could also trigger the exclusion of a person on social assistance. As mentioned in a previous section, several dentists were reluctant to perform treatments with a small profit margin, such as prosthetic care. This led some to “refer” people on social assistance to other professionals, such as denturists who, according to them, would have lower operating costs and thus better profit margins.

*So I refer social assistance cases to a denturist. He has an on-site lab. Because of that he is able to control lab costs and still offer a decent service to the patient in line with what the [government] can provide.* [Translation] [CL1]

## Discussion

Our study presents a series of strategies developed by dentists that have barely been described in the scientific literature. With respect to organisational issues (missed appointments), dentists develop strategies to maximise attendance, such as avoiding booking appointments in advance, and strategies to minimise the impact of non-attendance, such as booking two people at the same time. With respect to biomedical and financial issues, dentists have not found any satisfactory solutions and believe that it is up to the government to resolve these problems. Providing non-covered treatments for free, for instance, is a way to overcome a biomedical issue that, in counterpart, accentuates financial problems. Overall, dentists are reluctant to exclude people on social assistance but develop and apply solutions that discriminate against them.

Before discussing the findings in more detail, it is worth noting that our study reports the experiences and perspectives of a relatively small number of participants, even though the sample size is adequate, considering our qualitative approach [17]. On the contrary, the inductive nature of our methods provided data, the depth of which could hardly have been obtained through traditional quantitative research. Let us also mention that our sample did not comprise dental professionals who purposely and systematically exclude people on social assistance. It is probable that this practice, which has been observed in other contexts such as France [20] and the United States [5, 10, 21], also exists in Canada, as some of the participants acknowledged. Finally, we must point out that our findings may not be generalisable to dentists working in different contexts. Indeed, the problems encountered by dentists and the responses they provide depend not only on the characteristics of the dental care system – including public coverage, payment system, professional ideology, organisation of services – but also on societal values. Consequently, the findings may not apply to salaried dentists, for instance, who would not face the same financial issues as the study’s participants.

One important result is that even though participating dentists do not purposefully and systematically exclude people on social assistance, they may discriminate against them in different ways. A first kind of discrimination may occur at the start of the dentist-patient relationship and continue until the person on social assistance has demonstrated that he or she is a “good attendee”. During this initial “probation” period, some dentists, with the intent of maximising attendance and minimising the impact of non-attendance, selectively apply strategies to patients on social assistance. This is the case with the “double-booking” strategy, the advantages of which have already been discussed by Capilouto [22] but one that deserves further analysis. This strategy indeed constitutes a double-edged sword: on the one hand, it offers an opportunity for people on social assistance to access dental services and on occasion constitutes a last chance for those who have missed previous appointments; on the other hand, by potentially reducing the length of the clinical encounter, it does not favour a good therapeutic alliance, or high quality care. It is important to remember that previous research conducted among people on social assistance has underlined their many apprehensions about dental care [6, 7] and their need to be reassured [23]. In such a context, shortened appointments may frustrate people on social assistance and, by creating a “vicious circle”, even impede future attendance. Such potential “feed-back effect” has been suggested by Martin [24] with respect to medical care: poor relationship between patients and health professionals may lead to non-attendance, which would further weaken their relationship.

People on social assistance at times face a second and more discrete form of discrimination related to biomedical and financial issues. Dentists, who perceive these issues as a heavy burden, sometimes reduce the range of treatments that they present to people on social assistance. This not only applies to services that are not covered, such as endodontics, but also to those that are covered by the government, dentures in particular. This type of discrimination creates a “two-tier dentistry” situation, in which people receiving public coverage get minimal, low-cost services while more affluent people and beneficiaries of private insurance have access to more advanced care.

The two forms of discrimination that we have just described are contrary to the values of our society as mentioned by the 2002 Royal Commission on the Future of Health Care in Canada, namely equity, fairness and solidarity [15]. They also contradict three principles proposed by the American Dental Education Association to improve the oral health status of Americans [25]: “access to basic oral health care is a human right”; “the oral health care delivery system must serve the common good”; “the oral health needs of vulnerable populations have a unique priority”. In order to address these issues, an essential challenge of our time, we suggest reflecting on the following avenues of solution (Table 3).

**Table 3:** **Recommendations**

Types of issues	Our recommendations
Organisational (missed appointments)	Introduce drop-in periods (allow people to access dental services when ready or able to consult)
Biomedical (non-covered treatments)	Extend coverage to endodontic treatments
Financial (low fees)	Raise fees paid to dentists to an “acceptable” level
General issue	Reinforce dentists’ sensitivity to the situation of people on social assistance through the development of educational academic programs

First, with respect to organisational issues, our data suggest that the traditional model for appointments is not well suited to people living in poverty, who are often forced to live one day at a time because of their ongoing struggle for daily survival [26, 27]. George [28] concurs with this observation, noting that the appointment system is poorly adapted to people from socially deprived communities and can represent a barrier to health care. As a matter of fact, the solutions developed by participating dentists to improve attendance do not satisfy them very much, even though similar approaches, such as use of reminders and motivating patients, have shown some efficacy in medical care [28].

One solution would be to amend this traditional model with the introduction of drop-in periods, allowing people on social assistance and others to access dental services when ready or able to consult. It is interesting to note that immigrants in England suggested such a flexible approach in order to improve access to dental services [29]. Let us add that participants’ strategy of using people on social assistance as “fillers”, calling them at the last minute when a spot is available, somewhat acknowledges our suggestion. We should also consider more global strategies, such as “advanced access approaches”, which relies on the fact that demand is predictable. Even though we still lack information on their efficacy in dental care, they have shown promising successes in medical care [30, 31].

Second, we recommend raising the fees paid to dentists for treatments covered by public insurance to an “acceptable” level and extending the coverage to endodontic treatments, which would resolve a major dilemma faced by dentists. Let us remember that in the United States, among the 40 states that increased Medicaid payment rates between January 1997 and January 2000, 14 reported increases in dentist participation and/or utilisation of dental services [11]. A more recent study also showed that “higher Medicaid payment levels are associated with higher rates of receipt of dental care among children and adolescents” [32].

Finally, as many suggest [33–35], we recommend reinforcing dentists’ sensitivity to the situation of people on social assistance through the development of educational academic programs. We believe that “patient-centred” [36] and “social competency” approaches, for instance, can help professionals to better understand the perspectives of people living in poverty. These would facilitate the organisation of appointments by identifying appropriate time periods and avoiding the planning of patients’ unwanted visits. More generally, they could improve mutual understanding and prevent “damaged relationships between patients and practices”, a source of poor attendance [24].

## Conclusions

This study describes dentists’ efforts and solutions to keep people who are on social assistance within the dental care system, and their reluctance to exclude them. On the other hand, it shows that some solutions are often unsuccessful and other not sustainable in the long term. It also reveals that these strategies may discriminate against people on social assistance, which strongly contradicts professional and societal values. It is therefore necessary to address those issues by rethinking how dental services should be provided and financed.
